# A New Democratic Norm(al)? Political Legitimacy Amidst the COVID-19 Pandemic

**DOI:** 10.1007/978-3-030-65355-2_27

**Published:** 2021-03-20

**Authors:** Tim Reeskens, Quita Muis

**Affiliations:** 1 Tilburg University, Tilburg, The Netherlands; 2 Tilburg University, Tilburg, The Netherlands; 3 Tilburg University, Tilburg, The Netherlands; 4 Tilburg University, Tilburg, The Netherlands; Department of Sociology, Tilburg School of Social and Behavioral Sciences, Tilburg, The Netherlands

## Abstract

The worldwide COVID-19 pandemic has granted national governments far-reaching political powers to implement drastic non-pharmaceutical interventions to curtail the spread of the virus. For these measures to be effective, governments should be granted widespread political legitimacy. This is established when populations’ expectations from governments are in line with public support for these governments. In this chapter, we investigate changes in political legitimacy during the coronavirus crisis in the Netherlands. Amidst of the pandemic, we collected unique, representative data among LISS-panel respondents that supplemented the European Values Study 2017. We demonstrate that the Dutch public (temporarily) lowered their democratic aspirations thereby longing for strong leadership while simultaneously increasing their trust in the incumbent Government, which, combined, resulted in more political legitimacy. Because of an outspoken period effect, expectations are, however, that this legitimacy will not be long-lived in the new common.

With “patient zero” diagnosed at Tilburg’s ETZ hospital, the province of Noord-Brabant has been the bedrock to implement far-reaching non-pharmaceutical interventions to prevent COVID-19 from spreading across the Netherlands. The government response to COVID-19 was a self-proclaimed “intelligent lockdown,” which provided Dutch residents with relative freedom by allowing them to go outside but to do so responsibly (RIVM 2020). However, measures taken to “flatten the curve,” as advised by RIVM health experts, have unavoidably constrained some civil liberties too. The effectiveness of these interventions crucially depends on the public support not just for the measures but also for the political system implementing them. The aim of this contribution is to provide insights into how political legitimacy has changed amidst the COVID-19 pandemic by analyzing novel longitudinal panel data as part of the European Values Study.^1^

In this, the Netherlands is an interesting case as it is conceived of as a liberal democracy characterized by strong democratic appraisal (see Fig. 27.1; EVS 2020); furthermore, political institutions receive widespread public support (Bovens and Wille 2008). Political legitimacy^2^ combines these two aspects (Easton 1965; Norris 2011), namely expectations from the government (i.e., more democratic versus more authoritarian governance) together with support for their functioning. When populations have strong demands from the government, but the latter are unable to fulfill them, a “deficit” arises (Norris 2011). This deficit might ultimately jeopardize the successful implementation of required non-pharmaceutical interventions.

**Fig. 27.1:**
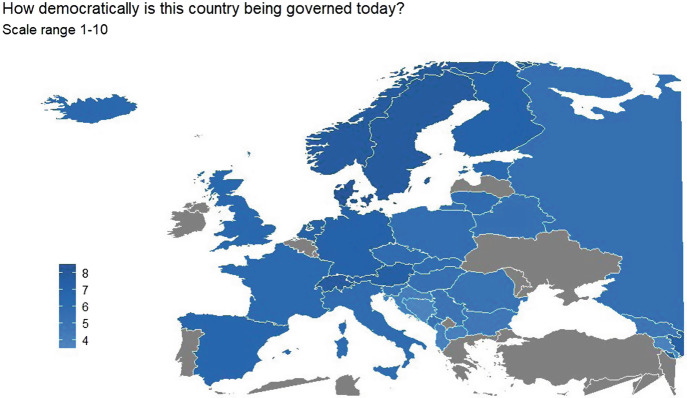
Perceptions of democratic governance across Europe, 2017 (source: European Values Study 2017 + own calculation)

At a more theoretical level, these public expectations from the government align with what is referred to as “diffuse system support.” This term represents a generalized attachment to the core values and principles of a political system (Norris 2011: 22), such as the separation of powers, freedom, self-determination, and moral autonomy (Dahl 1989). Evaluations of government functioning are also named “specific system support,” expressed by the popularity of and trust in, for example, incumbent prime ministers, party leaders, and political parties (Norris 2011: 21). Scholarship suggests that explanations for diffuse and specific support are largely different (Mishler and Rose 2001).

Diffuse support is thought to be rather stable because the attachment to more or less democratic values is understood to result from socialization at a young age (Inglehart 1977, 1997). Older generations were raised in insecure contexts, making them prioritize safety and stability while younger generations grow up in relative security, facilitating attachment to values such as individual freedom and autonomy. Psychological insights (Maslow 1943), however, suggest that in moments of crisis, when insecurity prevails, a short-term value change can occur. In such circumstances, people decrease their focus on civil liberties, thereby demanding stronger leadership (Boin and ‘t Hart 2003; Inglehart 1997).

By contrast, specific support often fluctuates as it depends on the functioning of incumbent political actors, which is usually based on a rational assessment of their performance, economic outcomes, and individual well-being (Norris 2011). In case of an existential threat, such rational evaluations make room for more emotional responses, such as the “rally round the flag effect” (Mueller 1973). Hence, political support is expected to increase during the crisis COVID-19 poses, regardless of the measures taken by the government.

## Analytical Strategy

To assess changes in diffuse and specific political support, we analyzed unique, representative panel data of the Dutch population as part of the European Values Study (EVS 2020) and fielded as part of the LISS Panel.^3^ Data were collected at two different time points: before (2017) and amidst (May 2020) the COVID-19 pandemic. For the initial data collection, 2053 respondents are interviewed, of which the majority (*N* = 1288) participated in 2017 and in the follow-up survey designed to capture the influence of the current pandemic. In this follow-up, several questions from the Main Questionnaire were repeated. Here, we are interested in people’s attitude towards having a strong leader who does not have to bother with parliament and elections (1 = very bad; 4 = very good) as measure for diffuse support; trust in government (1 = none at all; 4 = a great deal) is used as an indicator for specific support. These items are supplemented with unique questions measuring the perceived individual consequences of the coronavirus crisis; for this contribution, we include the item measuring salience of COVID-19, namely the extent to which people are generally concerned about the coronavirus crisis (1 = not at all; 5 = to a large extent). We also include time-invariant characteristics like age, gender, and education. After excluding respondents without information, 973 respondents remain.

To assess individual changes in diffuse and specific system support, we calculated a difference score by subtracting respondents’ answers in 2017 from their answers in 2020 (Allison 1990). We performed one-sample *t*-tests and regression analyses to arrive at the results described below. Post-stratification weights were applied.^4^

## Results

**Fig. 27.2:**
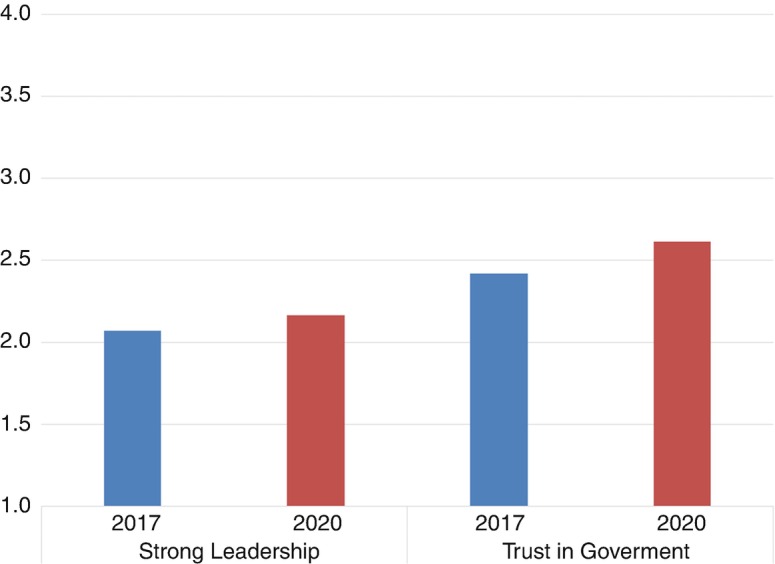
Change in political system support, 2017–2020 (source: European Values Study Netherlands 2017, 2020 + own calculations. Post-stratification weights are applied)

Figure 27.2 displays the overtime changes in diffuse and specific political support. For diffuse system support, we see an increase in the desire for a strong leader during the pandemic: the mean changed significantly from 2.07 in 2017 to 2.16 in 2020 (*p* < 0.01). For specific system support, we observe a significant increase in trust in government from 2.42 in 2017 to 2.61 in 2020 (*p* < 0.001). Together, the findings confirm our expectations that in times of crisis, people long for strong leadership instead of more democracy. At the same time, they evaluate the performance of political institutions more positively, which leads to more political legitimacy.

To explain these shifts, we find that the salience of the corona crisis does not lead to a demand for stronger leadership. However, those who are concerned about the crisis do indicate more trust in the Dutch government. While generation and gender turn out to be unrelated to diffuse system support, education does have an effect. Especially the lower educated increasingly prefer a strong leader. In addition, the higher educated have indicated more support for the government amidst the COVID-19 pandemic. Combined, although we observe some differences in the increase in diffuse and specific system support, political legitimacy has largely increased in society as a whole (Table 27.1).

**Table 27.1:** Changes in diffuse and specific political support regressed on relevant covariates, Netherlands 2017–2020 (Source: European Values Study Netherlands 2017, 2020)

	Support for strong leader	Trust in government
Intercept	1.650^***^ (0.157)	1.147^***^ (0.117)
Salience of COVID-19	0.017 (0.031)	0.056^*^ (0.023)
Generation (Ref: Great Gen)		
– Baby boom	–0.107 (0.081)	–0.079 (0.059)
– Generation X	–0.112 (0.088)	–0.091 (0.064)
– Millennials	–0.106 (0.089)	–0.010 (0.065)
Levels of education	–0.090^***^ (0.015)	0.029^**^ (0.011)
Gender (Ref: Man)		
– Woman	0.056 (0.052)	0.028 (0.038)
2017 level	–0.579^***^ (0.028)	–0.502^***^ (0.028)
R^2^	0.334	0.266

*p < 0.05; **p < 0.01; ***p < 0.001. Entries represent parameter estimates from two OLS regressions, with standard errors between brackets. Post-stratification weights are applied

## Conclusion

In this chapter, we have demonstrated how the Dutch government has gained political legitimacy amidst the COVID-19 pandemic through decreased democratic aspirations and, simultaneously, increased trust in government. These findings confirm the idea that, in times of crisis, people find comfort in strong leadership, thereby turning to illiberal tendencies, and that their rational evaluation of the government is, at least temporarily, replaced by a more emotionally driven “rally” effect. The fact that this is present in large sections of society indicates a specific and unique period effect.

The implementation of non-pharmaceutical interventions, and particularly the intelligent lockdown, required political legitimacy, and this appeared to have been successful. However, existing research already indicates that such shifts in political support in response to crisis situations are often temporary. Hence, it is important for national governments to be aware of the fact that the political legitimacy they have been enjoying can vanish sooner rather than later. Combined, although some authors have recently expressed optimism that the implementation of non-pharmaceutical interventions to contain the coronavirus has rejuvenated democracies (see Bol et al. 2020), our understanding is that such expectations of widespread political legitimacy in the new common are rather grim.

## Footnotes

1 We would like to thank Loek Halman, Ruud Luijkx, Inge Sieben and Leen Vandecasteele for valuable comments on this contribution, as well as Giovanni Borghesan for producing the post-stratification weights.

2 We understand “political legitimacy” from a Weberian perspective as it describes it by “the basis of every system of authority, and correspondingly of every kind of willingness to obey, is a belief, a belief by virtue of which persons exercising authority are lent prestige” (Weber 1964: 382).

3 The LISS Panel consists of approx. 7500 individuals, drawn from the population register based on a true probability sample, hence representative of the Dutch population. Each month, panel members are asked to respond to questionnaires. For more information, see http://www.lissdata.nl.

4 Due to word count limitations, we are not able to present more detailed information on the data, variables, and analyses. Please contact the first author in case of any questions.
